# Opposite Drug Prescription and Cost Trajectories following Integrative and Conventional Care for Pain – A Case-Control Study

**DOI:** 10.1371/journal.pone.0096717

**Published:** 2014-05-14

**Authors:** Tobias Sundberg, Max Petzold, Niko Kohls, Torkel Falkenberg

**Affiliations:** 1 Karolinska Institutet, Department of Neurobiology Care Sciences and Society, Division of Nursing, Research Group Integrative Care, Huddinge, Sweden; 2 I C – The Integrative Care Science Center, Järna, Sweden; 3 Centre for Applied Biostatistics, Occupational and Environmental Medicine, University of Gothenburg, Gothenburg, Sweden; 4 Division Integrative Health Promotion, University of Applied Sciences and Arts, Coburg, Germany; 5 Generation Research Program, Ludwig-Maximilians-University, Bad Tölz, Germany; 6 Brain, Mind & Healing Program, Samueli Institute, Alexandria, Virginia, United States of America; National Institutes of Health, United States of America

## Abstract

**Objectives:**

Pharmacotherapy may have a limited role in long-term pain management. Comparative trajectories of drug prescriptions and costs, two quality-of-care indicators for pain conditions, are largely unknown subsequent to conventional or integrative care (IC) management. The objectives of this study were to compare prescribed defined daily doses (DDD) and cost of first line drugs for pain patients referred to conventional or anthroposophic IC in Stockholm County, Sweden.

**Methods:**

In this retrospective high quality registry case-control study, IC and conventional care patients were identified through inpatient care registries and matched on pain diagnosis (ICD-10: M79), age, gender and socio-demographics. National drug registry data was used to investigate changes in DDD and costs from 90/180 days before, to 90/180 days after, index visits to IC and conventional care. The primary selected drug category was analgesics, complemented by musculo-skeletal system drugs (e.g. anti-inflammatories, muscle relaxants) and psycholeptics (e.g. hypnotics, sedatives).

**Results:**

After index care visits, conventional care pain patients (n = 1050) compared to IC patients (n = 213), were prescribed significantly more analgesics. The average (95% CI) group difference was 15.2 (6.0 to 24.3), p = 0.001, DDD/patient after 90 days; and 21.5 (7.4 to 35.6), p = 0.003, DDD/patient after 180 days. The cost of the prescribed and sold analgesics was significantly higher for conventional care after 90 days: euro/patient 10.7 (1.3 to 20.0), p = 0.025. Changes in drug prescription and costs for the other drug categories were not significantly different between groups.

**Conclusions:**

Drug prescriptions and costs of analgesics increased following conventional care and decreased following IC, indicating potentially fewer adverse drug events and beneficial societal cost savings with IC.

## Introduction

Swedish health bylaws state that every county council is obliged to provide residents with taxpayer-funded high-quality medical care and appropriate health promotion programs. Like many other countries, Sweden faces numerous challenges in relation to its conventional care system, including funding and securing quality of care as well as maintaining efficiency of health services. Recently there have been legal attempts to promote increased pluralism in the Swedish health care system [1], [2]. Internationally, integrative care (IC) systems have been recommended by the World Health Assembly and the Director General of the World Health Organization [3]. IC health services generally integrate insights from medicine, the humanities, ethics and philosophy in a person-centred health care model, which combines conventional care services with specific complementary systems and therapies, with the aim to achieve a pluralistic, accessible, affordable, safe and effective health system [4]. Today, IC has become a strategic but much debated hallmark of several national and international research and policy agendas [5]–[7]. Therapies such as massage, manual therapy, acupuncture and mindfulness can be found in current national care guidelines for managing persistent and recurrent disorders such as pain and depression [8], [9]. It is critical that this development adhere to clinically and cost-effective health care systems. Opponents argue that such developments are neither rational nor effective, and are effectively overburdening the healthcare system. Recently, emerging IC research findings have demonstrated evidence of significantly improved patient reported health outcomes such as self rated health, reduced pain and improved quality of life after anthroposophic IC [10], [11]. However, evidence of the comparative effectiveness of IC and conventional care health care models is urgently needed for evidence informed decision-making, and health technology assessments of IC have been called for [12].

In this study, we have used objective outcomes from high quality national registries in the Swedish health and medical services, to compare IC and conventional inpatient care for diagnosed pain patients, in terms of prescribed defined daily doses (DDDs) and cost of first line drugs, subsequent to index visits to IC and conventional care.

## Materials and Methods

### Ethics statement

The Regional Ethics Committee in Stockholm approved the study. Patient records/information was retrieved as anonymized and de-identified data from national and county council health care registries, thus no individually written informed consent procedures were conducted for this study. All data was kept anonymized and de-identified throughout the study and the results were only reported at group level.

### Design and setting

Retrospective case-control study.

### Conventional care management

The guideline for conventional care pain management in Stockholm County Council typically involves pain diagnosis and initiation of treatment by the patient's general practitioner where physical activity should be encouraged, sick leave kept to a minimum and the patient's work level maintained [13]. Referral to inpatient departments and pain rehabilitation clinics can follow to offer in-depth analysis of pain and relevant treatment options, including multimodal rehabilitation measures such as behavioural interventions, together with occupational and physical therapy interventions [13]. Pharmacotherapy ought to start with a low dose and titrate up with due regard for efficacy and possible side effects. Results stemming from short-term treatment trials with non-prescription analgesics should be considered, emphasizing that the pharmacological part of the treatment plan frequently has a limited role in the management of chronic pain conditions [13]. Sleep disorders developing as a consequence of the pain condition should be treated. While short-acting opioids preferably are to be avoided, pain specialists should be consulted for the potential deployment of long-acting opioids. All drug treatments should be evaluated after two months and discontinued if the maximum tolerated dose does not result in a clinically meaningful reduction in pain and/or increased function. If continued, pharmacotherapy should be reassessed at regular intervals [13].

### IC management

Conventional care providers in Stockholm County have been able to refer patients to anthroposophic IC since the 1980s. Anthroposophic IC is delivered by conventionally trained physicians, nurses and therapists who have additional training in anthroposophic medicine, whereby conventional care is integrated with selected complementary therapies such as massage, music and art therapy, natural remedies and diet/nutrition in an amplified healing environment [14], [15]. Internationally, anthroposophic IC is a prevalent form of health care service found at approximately 24 anthroposophic hospitals and 200 outpatient clinics with an estimated total of 2.700 fully trained licensed medical doctors and around 15.000 medical doctors with various levels of training [16].

### Patient observations and matching

IC and conventional care patient observations were identified through inpatient registry data from Stockholm County Council covering years 2005 to 2010. The observations were matched with regard to diagnosis, age (18–39 years, 40–49 years, 50–59 years, and 60+), gender (male/female) and socio-demographics (three classes High, Affluence and Low). The latter was achieved by utilizing the Mosaic geodemographic segmentation system employed in the Stockholm County Council health care registries [17]. In short, the Mosaic system makes use of multivariate statistical classification technique for categorizing the population into different socio-economic groups, based on relevant statistical variables [18]. Data used to build the Mosaic classification is primarily derived from Statistics Sweden with its high quality records that include the Directory of total population, the Real Estate Tax Register, the Income and Wealth Register, the Register of Education and data from Parliamentary Elections [18], [19]. Sweden is divided into 74 000 Mosaic areas, the smallest, most common size for which is 125×125 meters, which means that the accuracy in analyses can be very high without harming the integrity of the individual [18].

### Index visits and diagnosis

Index visits were defined as the first registered inpatient visit with the target pain diagnosis (ICD-10: M79). This selected diagnosis chapter entails “Other soft tissue disorders, not elsewhere classified” and typical pain disorders include myalgia, fibromyalgia, unspecified rheumatism and pain in the limbs [20].

### Measurements and data collection

The primary outcomes were prescribed and sold DDDs and the related costs of these drugs, which were analysed in two time periods before and after the index visit to IC or conventional care: average values from 90 days before/after, in total 180 days; and average values from 180 days before/after, in total 360 days. Data on drug prescriptions, DDD and costs in Swedish kronor were collected from the Swedish national drug registry that is maintained by the National Board of Health and Welfare (Socialstyrelsen), a Swedish government agency under the Ministry of Health and Social Affairs [21]. The cost of drugs was converted to euros using an approximate exchange rate of ten Swedish kronor to one euro. Drug categories were confined by identification of Anatomical Therapeutic Chemical classification codes relevant to first line drugs recommended in the management of pain, i.e. the primary selected drug category was analgesics (code N02, e.g. analgesics, opioids), complemented by musculo-skeletal system drugs (code M, e.g. anti-inflammatories, muscle relaxants) and psycholeptics (code N05, e.g. hypnotics, sedatives) [22], [23].

As a secondary measure, to describe the disease load/comorbidity burden of the IC and conventional care groups over time, we mapped the number of diagnostic categories (A–Z) registered per patient for the IC and conventional care groups during the year before and the year after the index visits.

### Statistics

Standard statistical procedures were employed for calculating means, standard deviations and confidence intervals. Data was visually checked for normal distribution and as no severe violation was detected in relation to sample size, submitted to parametric testing procedures. Paired and unpaired t-tests were used to test for differences between DDDs and costs of drugs for the IC and conventional care groups over time. All statistical tests were two-tailed and corrected for unequal group variances as necessary. The level of significance was 5%. Statistical software included SAS and STATA12.

## Results

### Patients and matching

In total there were 213 observed patients in the IC group with the target pain diagnosis. Given the available subgroup sizes for matching in the corresponding conventional care group we aimed to sample about 5 conventional care patients per IC patient. A final sample of 1050 matched control patients in the conventional care group was obtained. The observed sample characteristics are shown in Table 1.

**Table 1:** Patient characteristics, matching and baseline values for the integrative care and conventional care groups.

	Integrative care (n 213)	Conventional care (n 1050)
Matching % (n)		
Diagnosis ICD-10: M79	100 (213/213)	100 (1050/1050)
Female	100 (213/213)	100 (1050/1050)
Age:		
0–39 years	19 (41/213)	19 (200/1050)
40–49 years	32 (68/213)	30 (320/1050)
50–59 years	36 (76/213)	37 (390/1050)
60+ years	13 (28/213)	13 (140/1050)
Socio-demographic classification (Mosaic):		
High	48 (103/213)	49 (515/1050)
Affluent	19 (41/213)	20 (205/1050)
Low	31 (66/213)	31 (330/1050)
Missing	1 (3/213)	0
Baseline values		
Prescribed drugs (DDD/patient):		
ATC-M 90 days PRE	1.2 (0.0 to 2.3)	1.7 (1.1 to 2.4)
ATC-N02 90 days PRE	8.0 (4.4 to 11.6)	11.9 (8.3 to 15.5)
ATC-N05 90 days PRE	19.4 (12.5 to 26.3)	20.4 (16.1 to 24.7)
ATC-M 180 days PRE	2.7 (−0.1 to 5.6)	3.9 (2.6 to 5.1)
ATC-N02 180 days PRE	14.2 (8.1 to 20.4)	22.5 (16.0 to 28.9)
ATC-N05 180 days PRE	34.7 (23.1 to 46.3)	38.9 (31.5 to 46.2)
Cost of drugs (euro/patient):		
ATC-M 90 days PRE	7.7 (4.4 to 10.9)	9.7 (7.5 to 11.9)
ATC-N02 90 days PRE	24.0 (16.1 to 31.8)	25.1 (20.1 to 30.1)
ATC-N05 90 days PRE	14.9 (2.3 to 27.5)	11.6 (8.6 to 14.5)
ATC-M 180 days PRE	15.0 (9.5 to 20.4)	19.4 (15.7 to 23.1)
ATC-N02 180 days PRE	40.2 (28.6 to 51.8)	45.0 (36.6 to 53.4)
ATC-N05 180 days PRE	21.9 (7.1 to 36.7)	23.0 (17.3 to 28.8)

ICD-10, International Classification of Diseases version 10. ATC, Anatomical Therapeutic Chemical Classification System: ATC-M (Musculoskeletal system, e.g. anti-inflammatories and muscle relaxants; ATC-N02 (Analgesics); ATC-N05 (Psycholeptics). DDD, Defined daily dose. PRE, value preceding index visit. Average (95% confidence interval) values unless otherwise stated. Analyzes by t-tests (two-tailed). There were no statistically significant differences between groups at baseline.

### Drug prescriptions

After the index visits, the conventional care pain patients were prescribed significantly more analgesics compared to the IC patients (Tables 2 and 3). The average group difference for analgesics at 90 days post index visit was 15.2 (95% CI: 6.0 to 24.3; p<0.001) DDDs/patient. At 180 days, the group difference was 21.5 (95% CI: 7.4 to 35.6; p = 0.003) DDDs/patient.

**Table 2:** Drug prescriptions and cost of drugs during 90-days PRE/POST first observed inpatient visit with ICD-10 diagnosis M79 for the integrative care and conventional care groups.

	Integrative Care (n 213) Conventional Care (n 1050)		Conventional-Integrative
	Change from 90-days before to 90-days after index visit		Difference in change between groups
DDD/patient:			
ATC-M	−0.3 (−0.9 to 0.3), p = 0.345	0.7 (−0.2 to 1.5), p = 0.128	1.0 (−0.9 to 2.9), p = 0.320
ATC-N02	−3.7 (−7.3 to −0.1), p = 0.042	11.5 (7.4 to 15.5), p<0.001	15.2 (6.0 to 24.3), p = 0.001
ATC-N05	−3.3 (−8.0 to 1.5), p = 0.176	1.9 (−1.0 to 4.9), p = 0.200	5.2 (−1.7 to 12.1), p = 0.139
Euro/patient:			
ATC-M	0.4 (−2.6 to 3.3), p = 0.815	4.1 (1.9 to 6.5), p<0.001	3.8 (−1.4 to 9.1), p = 0.151
ATC-N02	−4.7 (−12.9 to 3.5), p = 0.258	6.0 (2.1 to 9.8), p = 0.003	10.7 (1.3 to 20.0), p = 0.025
ATC-N05	−1.2 (−6.7 to 4.3), p = 0.666	0.4 (−1.8 to 2.7), p = 0.699	1.7 (−4.0 to 7.3), p = 0.565

DDD, Defined daily dose. ATC, Anatomical Therapeutic Chemical Classification System: ATC-M (Musculoskeletal system, e.g. anti-inflammatories and muscle relaxants; ATC-N02 (Analgesics); ATC-N05 (Psycholeptics). Average (95% confidence interval), p values; Analyzes by t-tests (two tailed).

**Table 3:** Drug prescriptions and cost of drugs during 180-days PRE/POST first observed inpatient visit with ICD-10 diagnosis M79 for the anthroposophic integrative care and conventional care groups.

	Integrative Care (n 213) Conventional Care (n 1050)		Conventional-Integrative
	Change from 180-days before to 180-days after index visit		Difference in change between groups
DDD/patient:			
ATC-M	−0.9 (−2.7 to 1.0), p = 0.373	0.0 (−1.4 to 1.4), p = 0.982	0.9 (−2.3 to 4.0), p = 0.591
ATC-N02	−4.7 (−10.1 to 0.6), p = 0.085	16.7 (10.5 to 23.0), p<0.001	21.5 (7.4 to 35.6), p = 0.003
ATC-N05	0.5 (−9.2 to 10.3), p = 0.916	5.6 (0.2 to 11.1), p = 0.042	5.1 (−7.7 to 18.0), p = 0.434
Euro/patient:			
ATC-M	4.0 (−2.2 to 10.2), p = 0.207	4.4 (0.9 to 7.9), p = 0.013	0.4 (−7.8 to 8.7), p = 0.917
ATC-N02	1.0 (−11.5 to 13.5), p = 0.878	10.3 (4.1 to 16.6), p = 0.001	9.4 (−5.6 to 24.3), p = 0.218
ATC-N05	9.4 (−3.0 to 21.7), p = 0.137	1.8 (−2.1 to 5.7), p = 0.365	−7.6 (−17.8 to 2.6), p = 0.146

DDD, Defined daily dose. ATC, Anatomical Therapeutic Chemical Classification System: ATC-M (Musculoskeletal system, e.g. anti-inflammatories and muscle relaxants; ATC-N02 (Analgesics); ATC-N05 (Psycholeptics). Average (95% confidence interval), p values; Analyzes by t-tests (two tailed).

### Costs

The cost of the prescribed and sold analgesics was significantly higher for conventional care pain patients compared to IC pain patients at 90 days post index visit (Table 2). The average cost difference was euro 10.7 per patient (95% CI: 1.3 to 20.0; p = 0.025).

### Disease load/comorbidity profiles

The IC and conventional care groups had similar frequency patterns of registered diagnosis categories the year before and after the index visits to IC and conventional care, albeit that the target diagnosis category (ICD-10: M) displayed the largest change over time (Table 4).

**Table 4:** Number of unique ICD-10 diagnostic categories (A-Z) per patient based on registered visits 365-days PRE/POST first inpatient visit for M79 (index visit).

	Integrative Care (n 213)		Conventional Care (n 1050)		Conventional-Integrative
	356-days PRE	Change 365-days POST	365-days PRE	Change 365-days POST	Difference in change between groups
ICD-10 category:					
A	0.02	0.01	0.06	0.05	0.03
B	0.09	0.05	0.18	0.00	−0.04
C	0.09	0.13	0.29	0.27	0.14
D	0.14	−0.03	0.32	0.18	0.21
E	0.44	−0.08	0.85	0.03	0.10
F	2.43	−0.16	1.84	0.23	0.39
G	0.20	−0.06	0.30	0.08	0.13
H	0.25	0.07	0.30	0.07	0.00
I	0.22	0.11	0.81	0.15	0.04
J	0.45	0.00	0.66	0.03	0.04
K	0.41	0.03	0.56	0.05	0.02
L	0.25	−0.01	0.31	0.02	0.03
**M**	**3.50**	**−1.04**	**2.55**	**0.40**	**1.44**
N	0.39	−0.02	0.51	0.10	0.13
O	0.02	0.07	0.13	−0.05	−0.12
P	0.00	0.00	0.00	0.00	0.00
Q	0.02	0.04	0.02	−0.01	−0.05
R	1.77	−0.46	1.75	0.22	0.68
S	0.19	−0.03	0.40	0.03	0.06
T	0.15	−0.10	0.23	0.09	0.19
U	0.00	0.00	0.00	0.00	0.00
V	0.01	0.00	0.00	0.01	0.01
W	0.08	0.00	0.12	0.03	0.04
X	0.04	−0.02	0.09	−0.02	0.00
Y	0.02	−0.01	0.06	0.05	0.07
Z	1.48	0.00	1.81	0.54	0.54

ICD-10, International Classification of Diseases version 10. Main diagnostic category M (Diseases of the musculoskeletal system and connective tissue) in bold.

## Discussion

### Key findings

The prescribed and sold DDDs/patient of first line drugs generally decreased for IC pain patients and increased for matched conventional care pain patients up to 180 days after index care visit. The largest differences were observed for the analgesics drug category, which also includes opioid drugs, which were significantly lower for IC patients after both 90 and 180 days post index care visit. The costs of the prescribed and sold analgesics showed similar patterns where IC patients had significantly lower costs compared with conventional care patients 90 days after the index care visit. The disease load/comorbidity profiles were generally similar with few changes in diagnostic category frequencies over time, albeit that for the target pain diagnostic category the trend was a decrease for IC patients and an increase for the conventional care patients.

### Possible explanations and comparison of study results with other published work

A previous prospective clinical study has been published, from the same anthroposophic IC hospital as in the current study, with a mix of patients groups including those with chronic pain. It was reported that rehabilitation with anthroposophic IC was beneficial to patients by facilitating new attitudes towards change and improved lifestyle habits, which in turn was reasoned to improve patients' general health over time [24]. Additionally, our recent research findings demonstrate significantly improved health outcomes such as self rated health, reduced pain and improved quality of life for patients after anthroposophic IC [10], [11]. It is possible that patients, after having received IC, improve their health status and quality of life and have the ability to change their behaviour and develop healthier lifestyle habits, which may in turn reduce the need for prescription drugs to manage their pain. In support of this, preliminary findings show reduction in inpatient health care services visits for IC (data not shown). Intriguingly, this would be coherent with the county council clinical care guideline that pharmacotherapy frequently has a limited role in chronic pain management [13]. Nonetheless, conventional care treatments typically rely substantially on the use of pharmacotherapy in the management of non-malignant pain [13], [22], [25], [26]. This should be viewed in contrast to IC strategies that also integrate complementary means in the care of patients. Considering this, a prospective international multicentre study comparing conventional care and IC practice showed that anthroposophic IC had more favourable outcomes both in terms of lower prescription rates and less adverse drug reactions in the management of acute respiratory and ear infections [27]. Although that study did not target pain patients, it may provide important insights about outcome differences and practice patterns between conventional care and anthroposophic IC, which in turn may help explain our current study results. It has likewise been suggested that potential cost differences in favour of anthroposophic IC can mainly be explained by less drug prescriptions and fewer referrals with anthroposophic IC [28]. Potentially fewer adverse drug events and cost-effective management due to less use of prescription analgesics with IC may indeed be of utter importance for safe and effective long-term care of patients suffering from pain conditions. Emerging evidence from a Swedish randomized controlled trial of another IC model, notably in the management of patients with chronic back/neck pain, showed that IC was feasible to implement, enabled patients to rely less on prescription and non-prescription analgesics compared to conventional care, and empowered patients towards increased self care strategies [29]–[31]. Lastly, recent registry analysis findings from the Netherlands attest to possible differences in resource use and health outcomes between conventional care and IC practice, where patients managed by physicians with training in anthroposophic IC or other complementary therapies, had lower health care utilisation and lived longer compared to patients that received care from conventionally trained doctors [32].

Estimates from Europe and Sweden show that approximately 20% of the population suffer from chronic pain of moderate to severe intensity, adversely impacting quality of life and working conditions [33], [34]. The costs for managing these pain disorders are enormous. In Sweden alone, the yearly expenditure has been estimated to range between 87.5 to 300 billion Swedish kronor, i.e. approximately up to euro 32 billion annually [26], [35]. Of these, the majority of the costs, about 60 to 90%, were indirect costs, reflecting production loss followed by illness-related absence from work and early retirement [26], [35]. The findings of reduced pharmaceutical costs in the present study indicate that IC might help defray the high societal expenditures by offering more cost-effective drug management. Further research into the potential reductions of indirect costs should be prioritized to enable extrapolations to determine broad national costs savings.

### Limitations and future research directions

High-quality care registries provide unique possibilities with high external validity to investigate and compare different treatment strategies in the management of costly disorders such as chronic pain. However, despite that the current study utilised a standard diagnostic code criteria to define the target pain diagnosis; that patient observations were matched on the basis of data from high-quality inpatient care registries; and that data on drug prescriptions and costs were collected from the national standard drug registry; i.e. that the overall quality of the data was high; a major limitation was the lack of clinically related variables that could be balanced. It is therefore possible that additional data, for example: the actual treatment protocols and care for each patient before/after the index visits; data on the duration, frequency and intensity of pain for each patient; indirect utilisation and costs including occurrences and costs of adverse drug reactions or care, could further improve the current estimates of outcome differences between IC and conventional care pain management. Similarly, unmeasured clinical parameters such as potential selection or referral bias of patients may also have impacted on the current results despite that no statistically significant differences between groups at baseline were found.

Further, the selected pain diagnosis (ICD-10: M79) is comprehensive and does not refer to a specific pathoanatomical diagnosis, but rather defines so-called non-specific pain disorders, and hence there may be large heterogeneity among patients. Although this is applicable to both the IC and conventional care groups, future studies, especially prospective trials, may want to address such issues in order to create more homogeneous groups, e.g. by employing additional inclusion criteria or by including validated disorder specific questionnaires in combination with diagnostic codes and perhaps clinical examination and assessment. Possibly, the combination of high quality registry data and prospective interventional pragmatic study designs, can be viable and strategic options to inform health policy and evidence-informed health sector reform, promoting a clinically and cost-effective health care system for patients suffering from pain disorders.

Finally, the generalizability of the results in this study may be questioned considering the complex and individualized package of anthroposophic IC services delivered. However, the fully trained licensed medical doctors at the IC hospital in the current study (Vidarkliniken) are expected to follow the International Guidelines for Good Professional Practice in Anthroposophic Medicine [36] as well as conventional guidelines and hence the current findings are likely to be generalizable to other country contexts also. In addition to investigating the specific effects of the individual components of anthroposophic IC, future research should assess the level of generalizability and patient preferences, preferably by multi-center clinical trials across e.g. Europe.

## Conclusions

Drug prescriptions and costs of analgesics increased following conventional care and decreased following IC. The demonstrated outcomes indicate potentially fewer adverse drug events and beneficial societal cost savings following IC, considering current annual high costs for pain in Sweden. In general, referral of patients with relevant pain disorders to anthroposophic IC seems appropriate. Prospective explanatory clinical trials and future investigations including patient preferences and indirect costs are warranted.
